# Treatment of acromioclavicular dislocations with a concomitant coracoid fracture: a systematic review of 37 patients

**DOI:** 10.1016/j.jseint.2022.12.014

**Published:** 2022-12-29

**Authors:** Melle M. Broekman, Daniel E. Verstift, Job N. Doornberg, Michel P.J. van den Bekerom

**Affiliations:** a Department of Surgery and Perioperative Care, Dell Medical School at the University of Austin at Texas, Austin, TX, USA; b Faculty of Behavioral and Movement Sciences, Department of Human Movement Sciences, Vrije Universiteit Amsterdam, Movement Sciences, Amsterdam, The Netherlands; c Department of Orthopaedic Surgery, Shoulder and elbow unit, OLVG, Amsterdam, The Netherlands; d Department of Orthopedic Trauma Surgery, Universitair Medisch Centrum Groningen, Groningen, The Netherlands

**Keywords:** Acromioclavicular, Coracoid process, Fracture, Dislocation, Treatment

## Abstract

**Background:**

Acromioclavicular (AC) dislocations with a concomitant fracture of the coracoid process (CP) are rare and there is ambiguity on treatment options. This systematic review was performed to address the clinically relevant question: what are the shoulder functions, union rates, and expected time until return to daily life in patients with a dislocation of the AC joint with a concomitant CP fracture after (1) nonsurgical treatment, (2) sole fixation of the AC joint, and (3) fixation of both the AC joint and the coracoid process?

**Methods:**

Studies were identified by conducting an online. Thirty records met the inclusion criteria and were suitable for data extraction.

**Results:**

A total of 37 shoulders from 37 patients were included. Surgical treatment was provided to 22 out of 37 patients, and 15 patients had nonsurgical treatment. Out of the surgically treated patients, 12 patients were treated with a fixation of both the AC joint and the CP, 9 patients with a sole fixation of the AC joint, and 1 patient with a sole fixation of the CP.

**Conclusion:**

Existing literature does not indicate that one treatment option is superior, and more data are needed to guide evidence-based decisions on this rare injury.

Acromioclavicular (AC) joint dislocations account for 12% of all dislocations in the shoulder girdle and 8% of all the dislocations in the human body.^27^ Scapular fractures make up fewer than 1% of all fractures, with coracoid process (CP) fractures accounting for 3%-13% of all scapular fractures.^21^^,^^31^ Fractures of the CP with a concomitant dislocation of the AC joint are even more rare and only a few cases have been reported in the past literature. Orthopedic surgeons often fail to diagnose the CP fracture on routine radiographs when an AC dislocation is at hand due to marked foreshortening and projection over the acromion or scapular blade.^22^

As a result of the scarcity of these combined injuries, there is ambiguity in treatment recommendations, and even the choice for surgical vs. nonsurgical management remains a subject of debate.^29^^,^^35^ Some studies suggest that surgeons should opt for a nonsurgical approach,^3^^,^^22^^,^^35^ whereas others advise surgical treatment with either a fixation of both the AC joint and the CP,^19^ or a sole fixation of the AC joint.^1^^,^^12^^,^^19^

This systematic review was performed to address the clinically relevant question: what are the shoulder functions, union rates, and expected time until return to daily life in patients with a dislocation of the AC joint with a concomitant CP fracture after (1) nonsurgical treatment, (2) sole fixation of the AC joint, and (3) fixation of both the AC joint and the coracoid process?

The overall aim of this systematic review is to combine the scarce literature to guide surgeons in their clinical decision-making when treating these injuries.

## Materials and methods

This systematic review was performed according to the Preferred Reporting Items for Systematic Reviews and Meta Analyses guidelines.

### Search

A search strategy was created in collaboration with a clinical librarian from Onze Lieve Vrouwe Gasthuis, Amsterdam, The Netherlands. Studies were identified by conducting a search in PubMed/Medline, Embase, CINAHL, Web of Science, SPORTDiscus, and EMBASE from inception up to and including January 24, 2022. Synonyms for “acromioclavicular joint dislocation” and “coracoid process fracture” were combined with accessory index terms and adjusted for every database. Details of the search are supplied in Supplementary Appendix S1.

### Selection

Records were identified with a specified search for each database and duplicates were removed in EndNote X8 (Clarivate, London, United Kingdom). Subsequent to identification, 2 authors (M.B. and D.V.) independently screened potentially relevant articles based on titles and abstracts using Rayyan. After screening, full texts were retrieved and assessed for eligibility. Disagreement was resolved by discussion. Reference lists of included articles were manually checked for additional relevant articles.

### Inclusion and exclusion criteria

Randomized trials, observational studies, and case reports were eligible for this review. Articles describing the results of treatment of patients with an AC dislocation with a concomitant fracture of the CP were eligible for this review. Articles were included if patients sustained a dislocation of the AC joint with a concomitant fracture of the CP which was treated nonsurgically, surgically by fixating both the CP and the AC joint, surgically by solely fixating the AC joint, or surgically with a sole fixation of the CP. Patients were included if the AC joint separation was classified as a Rockwood type II injury or higher. All studies had to be written in English, German, or Dutch language.

There were no restrictions on age, follow-up length, or date of publication. Reviews, cadaveric, biomechanical studies, and studies that did not describe functional outcomes or surgical technique were excluded.

### Quality assessment

The quality of the case reports was assessed with the Strengthening the Reporting of Observational Studies in Epidemiology-checklist. Each article was judged as poor, fair, or good*.*

### Data extraction and synthesis

Data were collected in Microsoft Excel (Microsoft Corporation, Redmond, WA, USA). Demographics and outcome variables were manually extracted. Variables extracted were baseline characteristics, follow-up length, type of treatment for both the dislocation of the AC joint and the CP fracture, range of motion (ROM), union rate, complications, return to sport and daily life activities, fracture type and disruption of both the coracoclavicular ligament and AC ligament. ROM was categorized into restricted or nonrestricted movement according to the Constant-Murley Score.^7^

## Results

A total of 922 records were identified by the initial database search. After the removal of duplicates, 425 records were screened for eligibility and 62 records were selected for full review. Thirty records met the inclusion criteria and were suitable for data extraction. All included studies concerned case reports or case series.

### Cohort descriptions

A total of 37 shoulders from 37 patients were included. The mean follow-up length was 11.3 months. Surgical treatment was provided to 22 (59%) out of the 37 patients (Table I). Within the surgical cohort, 12 (55%) patients had surgery on both the AC joint and the CP, 9 patients (41%) on solely the AC, and 1 patient (5%) on solely the CP. Nonsurgical treatment was performed in the remaining 15 out of 37 (41%) cases.

**Table I:** Nonsurgical vs. surgical outcomes.

Variables	Nonsurgical (n = 15)	Surgical (n = 22)
Mean time to reach full ROM (% of patients that reached full ROM)∗	3.6 months (93, 3%)	7.8 months (100%)
Non-displaced avulsion fracture	14	19
Displaced avulsion fracture	1	3
Reported a union rate of the CP (% of patients)	8 (53%)	17 (77%)
Complete union of the CP (% of all the cases that reported a union rate of the CP)	8 (100%)	12 (71%)
Mean follow-up time	5.6 months	11.1 months
Mean time to full return to sport or daily life activities (% of patients that reached full return)	5.6 months (93.3%)	9.1 months weeks (100%)
Residual complaints (% of patients)	5 (33%)	3 (13%)

*CP*, coracoid process; *ROM*, range of motion.

∗ Value is displayed as a mean for continuous variables with normal distribution.

### Outcomes nonsurgical treatment

A total of 11 articles describing 15 patients who were treated nonsurgically were included.^5^^,^^6^^,^^8^^,^^14^^,^^20^^,^^24^, ^25^, ^26^^,^^33^^,^^35^^,^^38^ Twelve patients were treated with a sling and ROM exercises. One patient was treated with a Robert-Jones bandage,^6^ one patient with a shoulder brace,^35^ and one patient with a Watson-Jones bandage.^24^ Fourteen out of 15 patients regained full ROM, with a mean time of 3.6 months (range, 1-12) (Table I). Morioka et al^26^ reported 1 patient who continued to have a mild posterior instability of the AC joint after 42 months. All 6 articles describing a union rate of the CP reported full union based on radiographs at the last follow-up. The remaining 5 articles describing 6 patients did not specify the union rate.^5^^,^^6^^,^^20^^,^^24^^,^^33^ The mean follow-up time concerned 5.6 months (range, 1-42). Full return to sports and daily life activities was possible in 14 out of 15 cases (93%) with a mean time of 3.7 months (range, 1-12). One patient was not able to fully return to sports and daily life activities.^26^ Residual complaints occurred in 5 patients (33%) and included subcoracoid impingement and bursitis,^35^ aching in the AC joint,^25^ residual AC deformities^24^ and cosmetic deformities.^20^^,^^25^

### Outcomes surgical treatment

We included 20 articles describing 22 patients who were treated surgically.^1^, ^2^, ^3^, ^4^^,^^9^, ^10^, ^11^, ^12^^,^^16^, ^17^, ^18^, ^19^^,^^22^^,^^29^^,^^30^^,^^36^^,^^37^^,^^40^^,^^41^ Twelve patients (55%) underwent fixation of both the AC joint and the CP,^2^^,^^3^^,^^9^^,^^11^^,^^16^, ^17^, ^18^^,^^22^^,^^29^^,^^30^^,^^40^^,^^41^ 9 patients (41%) had a sole fixation of the AC joint,^1^^,^^4^^,^^10^^,^^12^^,^^19^^,^^36^^,^^37^ and 1 patient (5%) was treated with a sole fixation of the CP^25^ (Table II). The most frequently used methods for fixation of the AC joint included the use of a clavicular hook plate and K-wires. In the majority of the patients, the CP was fixated with a cannulated screw. The mean time to reach a full ROM was 7.8 months (range, 1-30). Kälicke et al^17^ did not report a time to reach a full ROM. Sharma et al^36^ reported a case with a Constant-Murley Score of 92 at 6-month follow-up. All other patients regained full ROM. Full union of the CP was reported in 13 patients (59%). A nonunion was reported in 4 cases (18%), and for the remaining 5 patients the union rate was none-specified. The mean follow-up time concerned 11.1 months (range, 1-36). Fifteen cases reported a time until full return to sports and daily life activities, with a mean of 9.1 months (range, 2-30). Residual complaints occurred in 3 cases (14%) and concerned mild impingement pain due to a hook plate,^1^ a mild subluxation of the AC joint,^37^ and a mild asymptomatic deformity of the AC joint.^9^

**Table II:** Outcomes of different surgical procedures.

Variables	Fixation AC joint + CP (n = 12)	Sole fixation AC joint (n = 9)	Sole fxation CP joint (n = 1)
Mean time to reach full ROM (% of patients that reached full ROM)∗	8.1 months (100%)	7.7 months (100%)	52 weeks (100%)
Non-displaced avulsion fracture	11	8	0
Displaced avulsion fracture	1	1	1
Reported a union rate of the CP (% of patients)	7 (58%)	9 (100%)	1 (100%)
Complete union of the CP (% of all the cases that reported a union rate of the CP)	6 (86%)	7 (78%)	1 (100%)
Mean follow-up time	11.8 months	10.4 months	12 months
Mean time to full return to sport or daily life activities (% of patients that reached full return)	10.1 months (100%)	7.1 months (100%)	12 months (100%)
Residual complaints (%)	0 (0%)	3 (33%)	0 (0%)

*AC*, acromioclavicular; *CP*, coracoid process; *ROM*, range of motion.

∗ Value is displayed as a mean for continuous variables with normal distribution.

### Outcomes of fixation of both the AC joint and CP

We included twelve articles describing 12 patients who were treated with a fixation of both the AC joint and the CP. The mean time to reach a full ROM was 8.1 months (range, 1-30). The patient described by Kälicke et al^17^ did not specify a time to reach a full ROM. Full union of the CP was seen in 6 out of 7 cases (86%) that reported a union rate. In 5 cases (38%), the union rate was none-specified. Duan et al^9^ reported 1 patient with a partial union of the CP. The mean time until full return to sports and daily life activities was 10.1 months (range, 2-30). In 5 patients, the time until full return to sports and daily life activities was none-specified. No residual complaints occurred in this group.

Seven patients were treated with clavicular hook plates and 6 patients with K-wires (Table III). In 8 out of the 12 patients in this cohort, the CP was fixated solely with a cannulated screw. In 3 patients,^18^^,^^29^^,^^30^ the CP was repositioned with K-wires and fixated with a 4 mm screw. Kälicke et al^17^ fixated the CP using both a screw and polydioxane cord. A mean time of 6.1 months (range, 1-18) to reach a full ROM was found in the group with hook plates vs. 9.1 months (range, 1.5-19) in the group receiving treatment with K-wires. The mean time until full return to sports and daily life activities was reported in 4 out of 7 patients who received treatment with a hook plate and concerned 8.5 months (range, 1-18). In the cohort receiving treatment with K-wires, all 6 cases reported a time until full return to sports and daily life activities, with a mean of 5.7 months (range, 3-8). In the group receiving a hook plate, 1 residual complaint occurred concerning mild impingement pain due to the hook plate. In the group receiving K-wires, a mild subluxation of the AC joint was seen on a radiograph.

**Table III:** Outcomes of Hookplate vs. K-wires in AC joint fixation.

Variables	Hookplate (n = 7)	K-wires (n = 6)
Mean time to reach full ROM (% of patients that reached full ROM)∗	6.1 months (100%)	9.1 months (100%)
Mean time to full return to sport or daily life activities (% of patients that reached full return)	8.5 months (100%)	5.7 months (100%)
Number of patients that reported a time to full return to sport or daily life activity	4 (57%)	6 (100%)
Residual complaints (%)	1 (11%)	1 (17%)

*AC*, acromioclavicular; *ROM*, range of motion.

∗ Value is displayed as a mean for continuous variables with normal distribution.

### Outcomes of sole fixation of the AC joint

Seven articles describing 9 patients who were treated with a sole fixation of the AC joint were found. The mean time to reach a full ROM was 7.7 months (range, 1.5-19). A non-complete union of the CP was found in 2 patients (22%).^10^^,^^19^ The mean time until return to sports and daily life activities was reported in 8 out of 9 cases with a mean of 7.1 months (range, 3-24). Residual complaints occurred in 3 patients (33%) concerning mild impingement pain due to a hook plate,^1^ subluxation of the AC joint,^37^ and a mild asymptomatic deformity of the AC joint.^10^

### Outcomes of sole fixation of the CP

One patient was treated with a sole fixation of the CP using nonabsorbable suture anchors.^25^ Originally, a complete AC separation was reported. The AC joint was not exposed during surgery. Full ROM was reached in 12 months. At 12 months follow-up, a full union of the CP was found and a full return to sports and daily life activities was possible.

## Discussion

Sole coracoid fractures comprise less than 1% of all fractures and a concomitant luxation of the AC joint is even more rare. Surgical vs. nonsurgical treatment options for this injury still remain subject of debate. To the best of our knowledge, we identified all reported patients to date, and thereby add to the literature in order to contribute to our understanding of this rare injury. Our results suggest that there is no superior treatment.

This study should be interpreted in the light of strengths and weaknesses: there were several limitations to this study. First, we found a limited number of 37 patients described in 30 articles. We believe this number is insufficient to provide evidence-based recommendations regarding the treatment for this injury. Since the combination of a dislocation of the AC joint with a concomitant CP fracture is rare, most articles concern case reports. Analysis in a larger patient population might yield different outcomes for both conservative and surgical treatment options. Secondly, several included records did not report all relevant outcome measures, leading to a heterogenous data set. For example, in 6 out of the 15 patients that were treated conservatively, no union rate of the CP was reported. Third, included articles range from 1973 up to 2022. One might argue that surgical procedures have changed throughout the years and may result in different outcomes. Recent articles on sole AC dislocations still show good anatomical and functional results after treatment with a hook plate,^32^^,^^39^ while new anatomical reconstruction techniques are gaining popularity and show promising results.^13^^,^^15^^,^^23^ Nonetheless, we decided to include older articles as well to provide a complete summary of the current available literature since the available data on this combined injury are limited. Fourth, we included studies that had notably longer times to reach a full ROM and full return to sport or daily life activities compared to the mean resulting in a widespread time to reach full ROM. Considering the limited number of patients, these outliers have a relatively large effect on the mean, skewing the results. Fifth, not enough information was provided to determine what grade of AC dislocations were being treated in each group. With lower grades (II and III) being more likely to be treated nonsurgically and higher grades (IV and V) to be treated surgically, it would be valuable to have more data on both surgical and nonsurgical treatment for these grades and their consecutive outcome measures to be able to provide more specific treatment recommendations.

Regarding the mode of treatment, our results suggest that patients treated nonsurgically have a slightly smaller chance to reach full ROM and fully return to sports and daily life activities, but also have a shorter time to reach a full ROM and return to sports and daily life activities compared to patients that were treated surgically. However, no clear treatment preference can be drawn from these results due to low numbers and the lack of significance. Our findings align with existing evidence showing that nonsurgical treatment in isolated AC dislocations has similar outcomes compared to surgical.^28^ A notable finding in our current study is that all described union rates within the nonsurgical cohort did report full union of the CP fracture. We think this can be partially explained by the number of non-displaced fractures. Finally, out of the recorded residual complaints within the conservative group, three were due to cosmetic deformities. Whilst it was not recorded as a residual complaint, surgery results in scars which may also be considered a cosmetic issue.

Considering operative treatment, we compared three surgical techniques: fixation of both the AC joint and CP, sole fixation of the AC joint, and sole fixation of the CP. Since there was only one patient with a sole fixation of the CP, this result cannot be generalized. Taking into account that the 2 CP fractures with a non-complete union concerned a displaced hinge fracture^1^ and a displaced comminuted fracture,^36^ our findings suggest that fixation of the CP in non-displaced fractures seems to have no benefit regarding the union of the fracture. Of note, when the CP does not advance to union, future surgeries involving the CP are presumably more complicated to perform due to an unstable CP. Literature shows that if ligament reconstruction is needed for future surgery and the CP is not suited as a point of fixation, the lateral clavicula is a complex alternative site and therefore fixation on the scapula should be considered.^34^The last notable finding is the complication rate of 33.3% in patients treated with sole fixation of the AC. However, these are only 3 patients, and considering that none of the complications involve the CP, no conclusions can be drawn from this. Our results suggest that conservative treatment of a displaced CP fracture might result in a nonunion. However, no recommendation can be made since no clinically significant data are available. Lastly, we compared the outcomes of fixating the AC joint with a hook plate vs. fixation by K-wires. Due to the small numbers of these two cohorts, we believe no conclusions can be drawn from this.

In this review, we summarized all available data on treating an AC dislocation with a concomitant fracture of the CP. Although this study has several limitations as mentioned above, and available data are limited, we believe it contributes to our current understanding of this rare injury pattern. Future studies should include bigger case series in order to collect more data on this rare injury. When enough data are available, this systematic review could be repeated with a larger cohort to be able to provide evidence-based direction.

## Conclusion

We found that existing literature does not indicate that one treatment option is superior, and that more data are needed to be able to provide evidence-based recommendations regarding the treatment of this rare injury pattern.

## Disclaimers

Funding: No funding was disclosed by the authors.

Conflicts of interest: The authors, their immediate families, and any research foundation with which they are affiliated have not received any financial payments or other benefits from any commercial entity related to the subject of this article.
