# Supplementary material for: Treatment of acromioclavicular dislocations with a concomitant coracoid fracture: a systematic review of 37 patients
Source: JSES Int. 2022 Dec 29;7(2):225–9. doi: 10.1016/j.jseint.2022.12.014 (PMC9998878; doi:10.1016/j.jseint.2022.12.014)
Supplement: Supplementary Appendix S1 [file mmc1.docx]

**Appendix 1: Search Log**

| **Search** | **Query** | **Results** |
| --- | --- | --- |
| #4 | Search: **(("Acromioclavicular Joint"[Mesh] OR acromioclavicular[tiab] OR AC-joint*[tiab]) AND ("Joint dislocations"[MESH] OR dislocat*[tiab] OR luxat*[tiab])) AND ("Coracoid Process"[Mesh] OR coracoid*[tiab])** Sort by: **Most Recent**  ("Acromioclavicular Joint"[MeSH Terms] OR "acromioclavicular"[Title/Abstract] OR "ac joint*"[Title/Abstract]) AND ("Joint dislocations"[MeSH Terms] OR "dislocat*"[Title/Abstract] OR "luxat*"[Title/Abstract]) AND ("Coracoid Process"[MeSH Terms] OR "coracoid*"[Title/Abstract]) | [233](https://pubmed.ncbi.nlm.nih.gov/?term=%28%28%22Acromioclavicular+Joint%22%5BMesh%5D+OR+acromioclavicular%5Btiab%5D+OR+AC-joint%2A%5Btiab%5D%29+AND+%28%22Joint+dislocations%22%5BMESH%5D+OR+dislocat%2A%5Btiab%5D+OR+luxat%2A%5Btiab%5D%29%29+AND+%28%22Coracoid+Process%22%5BMesh%5D+OR+coracoid%2A%5Btiab%5D%29&sort=date) |
| #3 | Search: **"Coracoid Process"[Mesh] OR coracoid*[tiab]** Sort by: **Most Recent**  "Coracoid Process"[MeSH Terms] OR "coracoid*"[Title/Abstract] | [1,692](https://pubmed.ncbi.nlm.nih.gov/?term=%22Coracoid+Process%22%5BMesh%5D+OR+coracoid%2A%5Btiab%5D&sort=date) |
| #2 | Search: **"Coracoid Process"[Mesh]** Sort by: **Most Recent**  "Coracoid Process"[MeSH Terms] | [129](https://pubmed.ncbi.nlm.nih.gov/?sort=date&term=%22Coracoid+Process%22%5BMesh%5D) |
| #1 | Search: **("Acromioclavicular Joint"[Mesh] OR acromioclavicular[tiab] OR AC-joint*[tiab]) AND ("Joint dislocations"[MESH] OR dislocat*[tiab] OR luxat*[tiab])** Sort by: **Most Recent**  ("Acromioclavicular Joint"[MeSH Terms] OR "acromioclavicular"[Title/Abstract] OR "ac joint*"[Title/Abstract]) AND ("Joint dislocations"[MeSH Terms] OR "dislocat*"[Title/Abstract] OR "luxat*"[Title/Abstract]) | [1,696](https://pubmed.ncbi.nlm.nih.gov/?sort=date&term=%28%22Acromioclavicular+Joint%22%5BMesh%5D+OR+acromioclavicular%5Btiab%5D+OR+AC-joint%2A%5Btiab%5D%29+AND+%28%22Joint+dislocations%22%5BMESH%5D+OR+dislocat%2A%5Btiab%5D+OR+luxat%2A%5Btiab%5D%29) |

**Databases Searched:**

- PubMed
- Embase
- CINAHL
- Web of Science
- Sportdiscus
